# Outcomes Following the Arthroscopic Chondro-Gide Repair of Osteochondral Defects of the Talus

**DOI:** 10.7759/cureus.92621

**Published:** 2025-09-18

**Authors:** Yousef Al-Khatib, Daniel Haddad, Adesina Adetokunbo, Bennet Aboagye, Karim Hussien, Raghubir Kankate, Radwane Faroug

**Affiliations:** 1 Orthopaedics, Royal College of Surgeons in Ireland, Dublin, IRL; 2 Trauma and Orthopaedics, Buckinghamshire Healthcare NHS Trust, Aylesbury, GBR; 3 Trauma and Orthopaedics, Stoke Mandeville Hospital, Aylesbury, GBR

**Keywords:** ankle and foot, ankle arthroscopy, #arthroscopy, chondro-gide matrix, olerud-molander ankle score (omas), orthopaedics surgery, osteochondral defect, talus osteochondral defect, visual analogue scale (vas) for pain

## Abstract

Background and objective

Osteochondral defects (OCD) of the talus are associated with pain and ankle joint dysfunction. Autologous matrix-induced chondrogenesis (AMIC) is one of the arthroscopic surgical techniques described for the treatment of talar OCD. There is scarce evidence regarding the use of Chondro-Gide or any other synthetic matrix for OCD of the talus. We aimed to investigate patient outcomes following Chongro-Gide repair of talar OCDs, along with the relationship between lesion diameter and patient outcomes.

Methods

A retrospective cross-sectional study was carried out, and patient outcomes were recorded. Olerud-Molander Ankle Score (OMAS) was documented for each patient during clinic follow-ups, as well as visual analogue scores (VAS). Both preoperative and postoperative OMAS and VAS scores were recorded at the 12-week postoperative follow-up. The other factors recorded were as follows: lesion size, site of OCD, postoperative complications, patient age, and gender.

Results

Sixteen patients were identified with isolated talus OCD. The mean preoperative OMFAS was 30 (range: 10-45) while the postoperative OMAS was 72.7 (range: 65-100, p<0.01). The mean VAS score was 7.85 preoperatively compared to 2.69 postoperatively (p<0.01). The average OCD diameter was 8.9 mm (range: 3-14). There was no statistically significant correlation between OCD lesion diameter and improvement in OMAS scores (p>0.05). The most commonly reported complication by patients was postoperative stiffness.

Conclusions

We observed positive outcomes following arthroscopic osteochondral lesion repair using AMIC Chondro-Gide. While there is a paucity of evidence on the use of AMIC to treat talar OCDs, our study adds to the growing evidence endorsing arthroscopic AMIC with Chondro for treating talar OCDs.

## Introduction

Osteochondral defects (OCD) of the talus are associated with pain and dysfunction in the ankle joint [1]. Medial OCDs tend to be more common than lateral [1]. While conservative treatment is generally preferred in children and adolescents, surgical treatment generally depends on lesion size and location [2]. Autologous matrix-induced chondrogenesis (AMIC) has been described in the treatment of OCD in various joints, including the knee [3]. Mosaicplasty, osteochondral autograft transplantation, and bulk osteochondral allografting are some of the other surgical techniques described for treating OCD of the talus [1,4]. The Chondro-Gide matrix has been used in OCDs of the knee and metatarsophalangeal joint [5,6,7]. The use of the Chondro-Gide matrix has not been extensively described in the literature for osteochondral defects of the talus. Our study aims to investigate the outcomes of arthroscopic Chondro-Gide repair of OCDs of the talus.

## Materials and methods

Study design and setting

A single-centre cross-sectional observational study was carried out at the Buckinghamshire Healthcare National Health Service (NHS) Trust in the United Kingdom.

Data collection

Patient records were reviewed, and information was collected from MRI scan reports as well as clinic letters and in-person clinic follow-up appointments. All patients were followed up for 12 weeks postoperatively. Primary outcome measures were visual analogue scores (VAS) and Olerud-Molander Ankle Score (OMAS) for each patient, both pre- and post-operatively, collected at the 12-week follow-up appointment. Secondary outcome measures were MRI-reported size of OCD, site of OCD, patient age, gender, and postoperative complications.

Inclusion and exclusion criteria

The inclusion criteria were as follows: all patients who underwent arthroscopic talus OCD repair using the AMIC Chondro-Gide technique between October 2023 and December 2024. The exclusion criteria were as follows: patients who did not attend their 12-week follow-up postoperatively, patients with ankle OCDs that were not on the talus, and patients with conservatively treated talus OCDs.

Statistical analysis

Statistical analysis involved a paired Student’s T-test to determine the statistical significance between preoperative and postoperative VAS pain scores as well as OMAS. Spearman correlation was used to determine the relationship between OCD lesion diameter and change in OMAS.

Surgical method

The patient was positioned supine at the end of the bed, with a thigh tourniquet and a sandbag under the ipsilateral buttock. Anteromedial and anterolateral portals were created, avoiding nerves and tendons. The ankle was distracted and inspected in a systematic method to assess and cross-match with preoperative imaging. Once the OCD was located, it was debrided down to bleeding bone and a smooth edge. Using a nano pic, microfracture was performed in the subchondral bone bed. The superior smooth surface of the Chondro-Gide patch was marked with a surgical pen and cut to a size matching the OCD, and placed on top of the prepared bed using a grasper. The Chondro-Gide patch needs to fit snugly into the OCD defect and not overhang. Once a good position was achieved, Tisseel glue was applied through the arthroscopic portal via needle and syringe. The glue was applied around the margins of the OCD and on top under arthroscopic vision.

Rehabilitation

Patients were kept non-weight-bearing for two weeks in a backslab. Thereafter, they were transferred into a plastic boot and kept non-weight-bearing for a further four weeks, but encouraged to perform passive and active range of movement exercises. After week six, patients were asked to be fully weight-bearing in normal footwear and referred to physiotherapy for range-of-movement, muscle-strengthening, and proprioception exercises.

## Results

Sixteen patients were identified with isolated talus OCD. Five of them were female (31.25%) and 11 were male (68.75%). The mean patient age was 43.9 years (range: 25-75 years). The mean preoperative OMAS was 30 (range: 10-45) while the postoperative mean OMAS was 72.7 (range: 65-100, p<0.01). The mean VAS score was 7.85 preoperatively compared to 2.69 postoperatively (p<0.01) (Tables 1, 2).

**Table 1:** Average OMAS and VAS scores preoperatively and 12 weeks postoperatively OMAS: Olerud-Molander Ankle Score; VAS: visual analog scale

Parameter	Preoperative	Postoperative	Average change
OMAS	30	72.7	43.1
VAS score	7.85	2.69	5.2

**Table 2:** Results of the paired Student's t-test comparing preoperative and postoperative OMAS and VAS scores OMAS: Olerud-Molander Ankle Score; VAS: visual analog scale

Parameter	T-statistic	P-value
OMAS	-6.810	1.875
VAS score	11.434	8.265

The mean OCD diameter was 8.9 mm (range: 3-14 mm). The mean improvement in OMAS was 43.1 (range: 10-90) while the mean OCD diameter was 8.5 mm (range: 3-14 mm). There was no statistically significant correlation between OCD lesion diameter and improvement in OMAS (p>0.05) (Table 3).

**Table 3:** Spearman's correlation testing the relationship between OCD diameter and improvement in OMAS OCD: osteochondral defect; OMAS: Olerud-Molander Ankle Score

Parameter	Correlation coefficient	P-value
Spearman's correlation	0.293	0.410

Postoperatively, five patients reported stiffness (31.25%), and one patient developed a deep venous thromboembolism (6.25%) (Table 4). For all patients, the indication for surgery was persistent pain despite conservative management.

**Table 4:** Postoperative complications

Complication	Number of patients	Percentage
Stiffness	5	31.25%
Deep venous thromboembolism	1	6.25%
Total with complications	6	37.5%
No complications	10	62.5%

## Discussion

This study demonstrates that arthroscopic application of AMIC Chondro-Gide matrix for OCDs of the talus results in significant short-term improvements in both pain and functional outcomes. At 12 weeks, patients experienced marked increases in OMAS and reductions in VAS pain scores, with low complication rates. Notably, no correlation was observed between lesion size and clinical outcome, suggesting that the effectiveness of this technique may be independent of defect diameter within the assessed parameters. Our findings are consistent with existing literature on AMIC in the ankle. Valderrabano et al. reported significant functional improvement at up to five years following arthroscopic AMIC, with American Orthopaedic Foot and Ankle Society (AOFAS) scores increasing from 60 to 91 and VAS decreasing from 5 to 1.1 [8]. Similarly, Kekeç et al. observed sustained outcomes using an AMIC technique, with improvements in AOFAS and VAS scores over an average follow-up of three years, although their method required a medial malleolar window for an autograft [9].

Studies using open or arthroscopic AMIC have also found limited correlation between lesion size and clinical outcomes [9,10], which aligns with our findings. However, many of these studies incorporate imaging - such as MOCART (Magnetic Resonance Observation of Cartilage Repair Tissue) scoring - to assess cartilage repair, which was not included in our study and should be considered in future research. Compared with microfracture alone, AMIC provides a biological scaffold that may promote more efficient and durable fibrocartilage regeneration [11]. While osteochondral autografts and allografts remain viable alternatives, they are more technically demanding and carry higher donor site morbidity [12]. Our technique offers a less invasive and reproducible solution, particularly for moderate-sized lesions.

This study is limited by its retrospective nature, small sample size, and short-term follow-up, which provided us with only limited details regarding the prognosis following complications like ankle stiffness. However, our results add to the growing evidence supporting arthroscopic AMIC with Chondro-Gide in managing talar OCDs.

## Conclusions

We demonstrated a statistically significant improvement in ankle pain and functional outcomes following arthroscopic Chondro-Gide repair of OCDs of the talus. There was no correlation between OCD diameter and patient outcomes, which contrasts with published literature. Larger studies with higher sample sizes are needed to further investigate the outcomes and efficacy of AMIC and Chondro-Gide matrix, specifically in treating OCD of the talus.
